# Supplementary material for: Identification of photoperiod-induced specific miRNAs in the adrenal glands of Sunite sheep (Ovis aries)
Source: Front Vet Sci. 2022 Jul 22;9:888207. doi: 10.3389/fvets.2022.888207 (PMC9354845; doi:10.3389/fvets.2022.888207)
Supplement: Supplementary file 1 [file Data_Sheet_1.ZIP › Supplementary file/miRDeep2 file.zip!/miRDeep2 file/Know_Structure/SPLP42A3/oar-mir-376a.pdf]

miRBase precursor : oar-mir-376a
Total read count : 98
oar-miR-376a-5p read count : 58
oar-miR-376a-3p read count : 37
remaining reads : 3

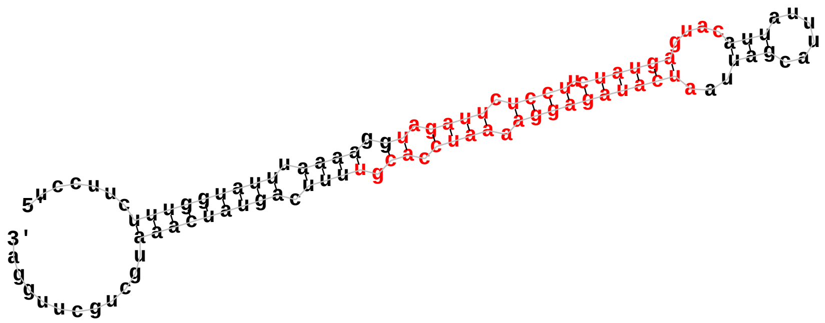

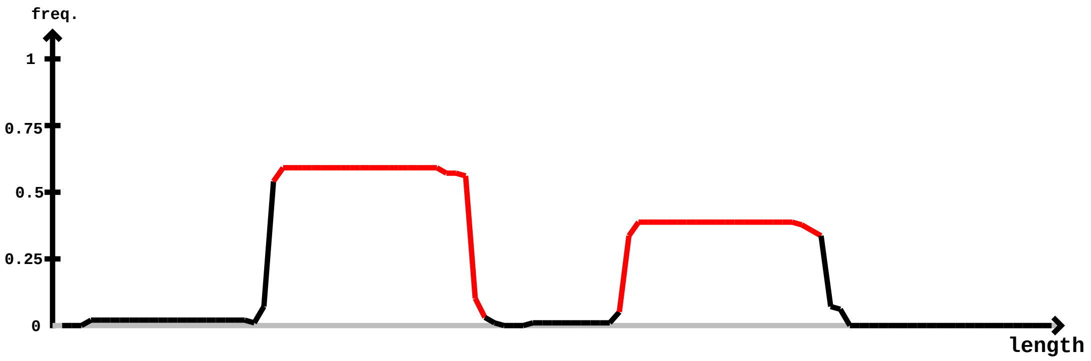

oar-miR-376a-5p

oar-miR-376a-3p

```
5'- uccuucuuugguauuuaaaagguagauucuccuucuaugaguacauuauuuacgauuaaucauagaggaaaauccacguuuucaguaucaaaugcugcuugga  -3'   exp
    ......((((((((.((((.((.((((.((((.((((((....(((......)))...)))))))))).)))).)).)))).)))))))).............   reads   mm   sample
    ..cuucuuugguauuuaaa....................................................................................   1       0    seq
    ..cuucuuugguauuuaaaag..................................................................................   1       0    seq
    ....................gguagauucuccuucuaug................................................................   1       0    seq
    ....................gguagauucuccuucuaugag..............................................................   1       0    seq
    ....................gguagauucuccuucuaugagu.............................................................   4       0    seq
    .....................guagauucuccuucuaugagu.............................................................   37      0    seq
    .....................guagauucuccuucuaugagua............................................................   7       0    seq
    .....................guagauucuccuucuaugaguac...........................................................   2       0    seq
    .....................guagauucuccuucuaugaguaca..........................................................   1       0    seq
    ......................uGgauucuccuucuaug................................................................   1       1    seq
    ......................uagauucuccuucuaugagu.............................................................   4       0    seq
    ................................................uuuacgauuaaucauagaggaaaauccacguuu......................   1       0    seq
    .........................................................aaucauagaggaaaauccacgu........................   4       0    seq
    ..........................................................aucauagaggaaaaucca...........................   1       0    seq
    ..........................................................aucauagaggaaaauccac..........................   2       0    seq
    ..........................................................aucauagaggaaaauccacg.........................   2       0    seq
    ..........................................................aucauagaggaaaauccacgu........................   22      0    seq
    ..........................................................aucauagaggaaaauccacguu.......................   1       0    seq
    ...........................................................ucauagaggaaaauccacguuu......................   5       0    seq
```
